# Post Covid telogen effluvium: the diagnostic value of serum ferritin biomarker and the preventive value of dietary supplements. a case control study

**DOI:** 10.1007/s00403-024-03004-1

**Published:** 2024-06-06

**Authors:** Nermeen Ibrahim Bedair, Alaa Safwat Abdelaziz, Fatemaalzahraa Saad Abdelrazik, Mohamed El-kassas, Mohamed Hussein AbouHadeed

**Affiliations:** 1 Department of Dermatology, Andrology, Sexual Medicine and STDs, Faculty of Medicine, Helwan University, Cairo, Egypt; 2 Department of Dermatology, Banha Educational Hospital, Ministry of Health, Banha, Egypt; 3 Department of Chest Medicine, Faculty of Medicine, Helwan University, Cairo, Egypt; 4 Department of Endemic Medicine, Faculty of Medicine, Helwan University, Cairo, Egypt; 5 Research Department of Dermatology and Venereology, Medical Research and Clinical Studies Institute, National Research Centre, Giza, Egypt

**Keywords:** Covid, Postcovid, Telogen effluvium, Ferritin, Biomarkers, Vitamin D, Vitamin C, Zinc, Lactoferrin, Ivermectin, Azithromycin

## Abstract

Telogen effluvium is characterized by excessive hair shedding usually following a stressful event. Ferritin has been used in clinical practice as a biomarker of nonanemic iron deficiency in cases of telogen effluvium. During the years of the COVID19 pandemic, telogen effluvium was reported as a part of post covid manifestations. As ferritin was also a biomarker for inflammation in cases with covid infection, this study was designed to evaluate the value of ferritin in cases with postcovid telogen effluvium one hundred patients recovering from covid 19 for 4–12 weeks were included in the study, detailed drug and laboratory history was obtained and serum ferritin level was measured. the mean serum level of ferritin among telogen effluvium patients was significantly lower than controls (68.52 ± 126 and 137 ± 137.597 ug/L respectively). Patients with telogen effluvium used significantly more azithromycin and ivermectin and significantly less vitamin C, D, lactoferrin and zinc than the controls Although serum ferritin is lower among telogen effluvium patients, it was still higher than the cutoff value for diagnosing nonanemic iron deficiency, we suggest that it will not be a good biomarkers in these cases. Our secondary outcomes showed that dietary supplements used during active infection such as vitamin C, D, lactoferrin and zinc might have a preventive value on postcovid hair loss, while azithromycin and ivermectin could have a negative long term effect on telogen effluvium.

## Introduction

Telogen effluvium (TE) is characterized by diffuse hair shedding that usually starts 2-3 months following a certain stressful event. It has been suggested that the precipitating event causes premature transition of the anagen follicles into catagen and telogen phases, resulting in excessive hair shedding. Stressors that can cause TE include delivery, psychological stresses, acute illness, fevers, hospitalization, surgeries, prolonged malnutrition, and certain medications^1^. Serum ferritin was often used as a biomarker in cases with TE. Several studies have reported serum ferritin deficiency in the TE patients [2–4].

The COVID-19 pandemic was associated with many of these stressors; those who were infected with the virus were under immense physical and psychological stress that was reported to affect patients for up to 12 months after recovery [5].

Post-COVID-19 TE is a frequent, usually self-limiting dermatological condition, reported to be reversible within 6–12 months post-infection in most cases. However, it has major aesthetic, psychological, and social implications among the general population, especially women [6].

Several biomarkers have been found to be associated with COVID 19 infection as well as its sequelae [7]. Certain cytokines were markedly elevated in severe cases [8] .

Serum ferritin levels have usually been used to determine iron reserves along with being an acute phase reactant [9]. In cases with COVID 19 infection; serum ferritin levels were reported to be elevated with their elevation associated with the severity of the condition and can be used as a predictor of severity/mortality [10].

Because of the lacking evidence on the serum ferritin levels in cases with post-covid hair loss, this study was designed to compare serum ferritin among patients recovering from COVID 19 with and without TE.

## Patients and methods

This observational case-control study included 100 patients recovering from COVID-19 recruited from the Dermatology outpatient clinic of Badr university hospitals, between August 2021 and June 2022 The study was approved by the Research Ethical Committee, Faculty of Medicine, Helwan University (REC-FMHU 46/2021), and fulfilled all the ethical aspects required in human research. All patients received full information about the study design and possible side effects. All recruits provided an informed consent to participate in the study.

The sample size was calculated based on previous studies [11]. who found that the adjusted the mean significant difference in the Serum ferritin level in case group was (40.4 ± 35.6) to control group. The sample size will be calculated using the following formula:

$$n=2{\left[\frac{\left({Z}_{\alpha /2} +{ Z}_{\beta }\right) * \sigma }{{\mu }_{1}-{\mu }_{2}}\right]}^{2}$$

Where: n = sample size, Zα/2 = 1.96 (The critical value that divides the central 95% of the Z distribution from the 5% in the tail), Zβ = 0.84 (The critical value that separates the lower 20% of the Z distribution from the upper 80%), σ = the estimate of the standard deviation of the mean Serum ferritin level in case group = = 40.4, µ1 = mean in the Serum ferritin level according to symptoms = 289.3, and µ2 = mean in Serum ferritin level in control group = 24. 6.

So, by calculation, the sample size will be equal to a total of 100 patients in both groups.

We included all patients over 18 years of age with history of COVID-19 infection that was confirmed by PCR testing 4–24 weeks prior to enrollment. Fifty patients complaining of hair shedding (TE group) and 50 patients with no history of hair shedding following COVID infection (control group). We excluded patients younger than 18 years old, patients with no confirmed COVID infection or with fist symptom of COVID starting less than 4 weeks or more than 24 weeks prior to enrollment. Patients with any preexisting hair disorder e.g. androgenetic alopecia, alopecia areata, trichotillomania and scarring alopecia, patients with any other dermatological condition, patients with preexisting systemic disease e.g. thyroid disorders, inflammatory bowel disease, etc. we also excluded patients on any systemic medications that causes hair loss within 6 months prior to enrollment e.g. chemotherapy, cimetidine, antithyroid drugs, amphetamines, bromocriptine, levodopa and tricyclic antidepressants (e.g., amitriptyline). Patients with very short hair were also excluded.

All participants were subjected to full medical and dermatological history taking before a thorough dermatological examination. The diagnosis of TE was made by typical history of excessive hair shedding (e.g., reduction of the ponytail in diameter, clogging of the shower drain by hairs) and following physical findings: positive pull test, diffuse or bitemporal thinning, and absence of anisotrichosis in trichoscopy. Hair pull test was performed by firmly pulling about 40–60 hairs between two fingers and positive test is confirmed when pulling out of 4–6 hairs or more. Anisotrichosisi in trichoscopy (> 10% miniaturized hair) was deemed compatible with androgenetic alopecia (AGA).

Serum ferritin measurement: Venous blood samples (5 ml) were withdrawn from all participants. Samples were centrifuged for 20-min at the speed of 2000–3000 r.p.m. and supernatant was removed. The serum was separated and stored immediately at -20 °C in the laboratory. After collecting serum samples from all participants, ferritin levels were measured using chemiluminescence method in the Beckman DXI800 (Beckman Coulter Unicel DXI800, CA, USA) with a normal reference range of 11 ng/mL − 306.8 ng/ml.

Statistical analysis: Data were analyzed using IBM SPSS (Statistical Package for Social Science) Statistics for Windows, Version 20.0. Armonk, NY: IBM Corp.). Data were statistically described in terms of mean ± standard deviation ± SD), median and range, or frequencies (number of cases) and percentages when appropriate. Numerical data were tested for the normal assumption using Kolmogorov Smirnov test. Comparison of numerical variables between the study groups was done using Student t test for independent samples. For comparing categorical data, Chi-square (χ^2^) test was performed. Exact test was used instead when the expected frequency is less than 5. Multivariate logistic regression analysis was performed to determine the effect modification of age on the effect of sF on the groups. Two-sided p values less than 0.05 was considered statistically significant.

## Results

**Fig. 1:**
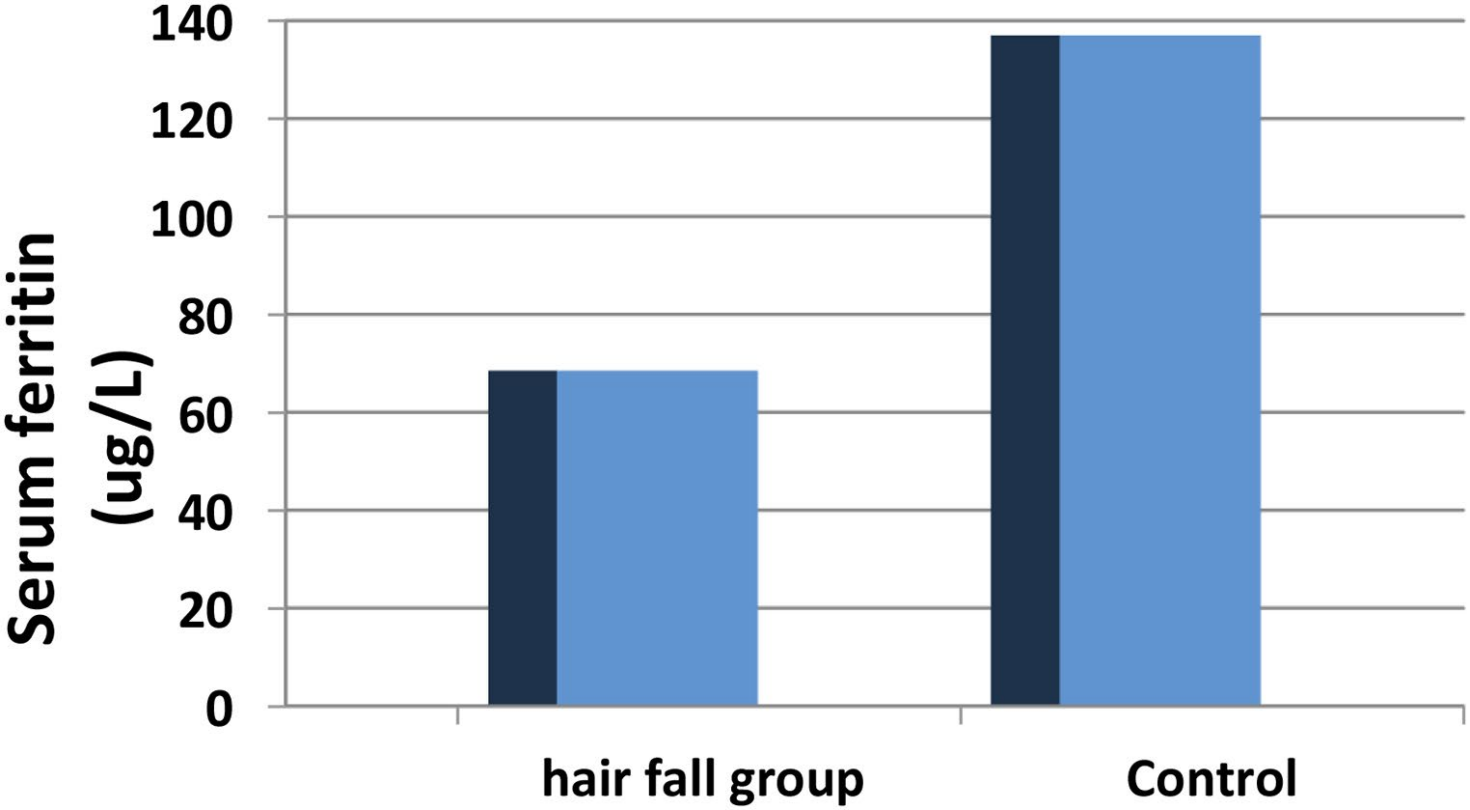
Serum ferritin level among TE patients compared to controls

one hundred patients recovering from COVID-19 were included in this study, 50 patients had post-covid telogen effluvium and 50 patients had no history of TE. After infection. Both groups had matching duration since at the time of enrollment (*p* = 0.880). TE patients were significantly younger (the mean age was 27.64 ± 6.04 and 33.84 ± 8.68 respectively *p* < 0.001) with significantly more females than the control group (*p* < 0.001) (Table 1). Serum ferritin level was significantly lower among TE patients than controls (68.52 ± 126 and 137 ± 137.597 ug/L respectively) (*p* < 0.001). Figure 1.

**Table 1:** Demographics of the studied patients

		Group		X^2^	*P*-value
		Hair fall *N* = 50	Control *N* = 50		
Gender	Female	48 (96%)	31 (62%)	17.42	< 0.0001
	Male	2 (4%)	19 (38%)		
Age	Mean ± SD	27.64 ± 6.04	33.84 ± 8.68	4.14	< 0.0001
	Median	27	32		
	Min- Max	18–45	18–55		
Duration since diagnosis (Weeks)	Mean ± SD	13.10 ± 5.76	12.92 ± 6.15	0.151	0.880
	Median	12	13		
	Range	4–28	4–24		

Comparing the use of medications and supplements for COVID-19 infection, the TE patients used significantly more azithromycin and ivermectin than the control group. On the other hand, the TE group consumed significantly less supplements of vitamin C, vitamin D, zinc and lactoferrin Table 2.

**Table 2:** medications used by both groups

		Group		*P*-value
		Hair fall *N* = 50	Control *N* = 50	
Azithromycin	No	13 (26%)	35 (70%)	< 0.0001
	Yes	37 (74%)	15 (30%)	
Ivermectin	No	24 (48%)	38 (76%)	0.004
	Yes	26 (52%)	12 (24%)	
Paracetamol		50 (100%)	50 (100%)	-
Vit-C	No	36 (72%)	15 (30%)	< 0.0001
	Yes	14 (28.0%)	35 (70%)	
Vit-D	No	48 (96%)	36 (72%)	0.001
	Yes	2 (4%)	14 (28%)	
Zinc	No	33 (66%)	8 (16%)	< 0.0001
	Yes	17 (34%)	42 (84%)	
Lactoferrin	No	44 (88.0%)	21 (42%)	< 0.0001
	Yes	6 (12%)	29 (58%)	

## Discussion

In the current study on 100 patients recovering from COVID-19, patients with TE had significantly lower serum ferritin levels and consumed significantly more azithromycin and ivermectin and less vitamin C, D, zinc and lactoferrin than patients who didn’t suffer from TE.

In the current study, patients with TE were significantly younger than controls and the mean age of the TE group was 33.84 ± 8.68 years. This was consistent with the study by Jubair et al., evaluated the role of ferritin levels with hair loss among female patients with COVID-19. They reported that the mean age of the patients was 27.58 ± 8.6 years which was significantly younger than the controls in their study (*P* = 0.011) [12]. Similarly, Babaei et al., found that the mean age of the patients was 30.97 ± 9.592 years [13], in contrast to these findings, in the study of Seyfi et al., the TE was more prevalent around the sixth decade of life [14].

In the present study, the duration since diagnosis of COVID infection till the time of presentation with hair fall in the patients group ranged from 4 to 28 weeks (1–4 months) with a mean value of 13.10 ± 5.76 weeks. This was consistent with most of the previous studies on post-covid TE., where they found that TE usually starts around months following covid infection [12, 13, 15–19]. Some few studies reported that TE might emerge as early as 1–2 months [6]. **O**ther studies reported that TE could start 3 months or more post-infection [14, 20, 21].

Several hypotheses suggested the etiology of post-covid TE. It was thought to be caused by high fever, as temperature might affect the hair cycle [22]. Our result didn’t validate this hypothesis as all our patients were received paracetamol with no significant difference between patients with TE and covid patients who didn’t experience TE. This was also suggested by other authors who suggested that as antipyretics don’t change the TE outcome, temperature is not the root cause of post-febrile TE and that inflammatory cytokines could be the main etiology [23] The inflammatory cytokines in covid, including interleukin-6 IL-6, tumor necrosis factor alpha TNFα, IL-1β, and interferon gamma IFNγ [24], were suggested to develop the catagen cycle in experimental studies [25, 26]. Furthermore, there is an interplay between cytokines and coagulation in covid infection [27] suggested to cause microthrombi formation which might obstruct hair follicle blood supply [26].

In the present study, serum ferritin level was statistically significantly lower in hair fall group than controls (*P* < 0.05).

This is in line with a previous study comparing women recovering from covid with versus without TE, Serum ferritin levels were also significantly lower among TE patients compared to the control group [28]. In contrast to other studies that found that post-COVID TE patients had no significant differences in ferritin levels compared to the controls [12, 29].

The discrepancy in laboratory parameters in TE patients with and without previous COVID-19 infection can be attributed to several factors: differences in sample size and patient characteristics with varying demographics, disease severity, and comorbidities compared to the current study. Variability in patient populations can influence the distribution of laboratory parameters and the likelihood of detecting significant differences. Differences in the timing of laboratory parameter measurements, assay sensitivity, and criteria used for defining TE and COVID-19 infection status can contribute to discrepancies between studies.

Although the serum ferritin was significantly lower among TE group among our patients’ cohort, it’s mean level was 68.52 ± 126 µg/L, this was high compared to the cutoff serum ferritin suggested by other authors as the minimal adequate level for serum ferritin (40 µg/L)[ 2–4,^30^,^31^]. We suggest that although our findings showed significantly lower serum ferritin level among TE patients compared to controls, it will not be a helpful biomarker for non-anemic iron deficiency in cases of post covid TE as the level is still higher than patients of TE without covid. As ferritin if an acute phase reactant [32], this might be attributed to the prolonged inflammation following covid infection. This is supported by the rapid elevation of ferritin levels in covid patients’ sera and suggests that it’s role might be pathogenic rather than an inflammatory biomarker [33].

In the current study, covid patients who used Vitamin-D supplements reported significantly less cases of TE than those who didn’t. Previous research found that vitamin D level was significantly lower among recovering covid patients who had TE than covid patients with no TE [28] In a previous case series on COVID patients with TE, 24% had vitamin D deficiency [13] This could be attributed to the fact that vitamin D plays a major role in stimulating immunity hence minimizing the toxic effect of covid on the hair follicle.

The favorable effect of vitamin D supplement on post-covid TE might be attributed to stimulatory effect of vitamin D on keratinocytes differentiation as the latest they express vitamin D receptor (VDR) [34]. VDR expression is highest during the anagen phase [35].

In the current study, patients with TE consumed significantly less zinc and lactoferrin supplements than the controls. The finding also reported previously [36]. Zinc is a potent promoter for proliferation of dermal papilla hence associated with hair follicle recovery. Dysregulation in zinc metabolism was implicated in several disorders of hair loss, including TE [37, 38]. Both zinc and lactoferrin supplements were widely used during the pandemic [39] and was recommended for cases of TE with serum zinc deficiency [40]. We attribute its favorable effect of lactoferrin against post covid hair loss by its ability promote the proliferation of dermal papilla [41].

On the other hand, patients with TE who were prescribed azithromycin and ivermectin significantly more than the control group. The role of azithromycin in post covid hair loss was previously suggested by other authors [42, 43]. To our knowledge, there was no previous studies on the effect of ivermectin treatment on post covid TE, however, ivermectin used during the pandemic was suggested to be ototoxic [44]. We suggest that both medications could have direct toxic effect on the hair follicle.

This study had several methodologic and scientific strength points. The case-control design with adequate sample size calculation was used to evaluate the mean ferritin level difference in TE patients recovering from covid. Detailed review of medications and supplements added more insights.

However; some limitations should be in mind when interpreting our results. A core limitation of the case-control design is that both causation and confounding are of potential concern.

**In conclusion**, the current study suggests that ferritin can be significantly lower in sera of post covid TE patients than patients recovering from covid without TE, but its level is still higher than the cutoff of serum ferritin level used in clinical practice to measure nonanemic iron deficiency in TE patients, hence we suggest that ferritin will not be as accurate biomarker in post covid TE. Our secondary outcomes revealed that dietary supplements used during covid could have a favorable effect on post covid TE.

## Data Availability

No datasets were generated or analysed during the current study.

## Abbreviations

TE: Telogen effluvium
